# A new assessment method for right ventricular diastolic function using right heart catheterization by pressure‐volume loop

**DOI:** 10.14814/phy2.15751

**Published:** 2023-07-02

**Authors:** Yoshitaka Isotani, Eisuke Amiya, Masaru Hatano, Hiroyuki Kiriyama, Masae Uehara, Junichi Ishida, Masaki Tsuji, Chie Bujo, Koichi Narita, Satoshi Ishii, Nobutaka Kakuda, Shun Minatsuki, Hiroki Yagi, Akihito Saito, Genri Numata, Takanobu Yamada, Takahiro Kurihara, Tatsuya Suzuki, Issei Komuro

**Affiliations:** ^1^ Department of Cardiovascular Medicine, Graduate School of Medicine The University of Tokyo Bunkyo‐ku Japan; ^2^ Department of Therapeutic Strategy for Heart Failure, Graduate School of Medicine The University of Tokyo Bunkyo‐ku Japan; ^3^ Department of Advanced Medical Center for Heart Failure, Graduate School of Medicine The University of Tokyo Bunkyo‐ku Japan; ^4^ Electrical Engineering Program, Graduate School of Science and Technology Meiji University Kawasaki Japan

## Abstract

Diastolic stiffness coefficient (β) and end‐diastolic elastance (Eed) are ventricular‐specific diastolic parameters. However, the diastolic function of right ventricle had not been investigated sufficiently due to the lack of established evaluation method. We evaluated the validity of these parameters calculated using only data of right heart catheterization (RHC) and assessed it in patients with restrictive cardiomyopathy (RCM) and cardiac amyloidosis. We retrospectively analyzed 46 patients with heart failure who underwent RHC within 10 days of cardiac magnetic resonance (CMR). Right ventricular end‐diastolic volume and end‐systolic volume were calculated using only RHC data, which were found to be finely correlated with those obtained from CMR. β and Eed calculated by this method were also significantly correlated with those derived from conventional method using CMR. By this method, β and Eed were significantly higher in RCM with amyloidosis group than dilated cardiomyopathy group. In addition, the β and Eed calculated by our method were finely correlated with E/A ratio on echocardiography. We established an easy method to estimate β and Eed of right ventricle from only RHC. The method finely demonstrated right ventricular diastolic dysfunction in patients with RCM and amyloidosis.

## 1 INTRODUCTION

There are several existing methods to assess right cardiac function, which include noninvasive methods, such as echocardiography and cardiac magnetic resonance (CMR) imaging. Unfortunately, they are often influenced by several confounding factors, such as preload and afterload, and may not reflect ventricular‐specific function (Lang et al., 2005; López‐Candales et al., 2006; Valsangiacomo Buechel & Mertens, 2012). Right ventricular (RV) function, like left ventricle, is involved in the course of heart failure. In particular, not only systolic function but also RV diastolic function has been reported to be related to pathological conditions, especially in the heart failure with preserved ejection fraction region (Jung et al., 2022; Lam et al., 2009). However, the evidence is limited due to insufficient evaluation methods.

Right heart catheterization (RHC) is one of the most reliable tests for hemodynamic evaluation in heart failure patients, and it greatly assists in determining treatment strategies in clinical practice (Binanay et al., 2005; Connors Jr et al., 1996; Sandham et al., 2003). However, the usage of RHC data had been generally limited, and there should be more sufficient studies analyzing RHC data including RHC waveform.

In terms of understanding hemodynamics, the pressure‐volume loop (PV loop), which is formed by simultaneously measuring the pressure and volume in the ventricle and plotting them in the same plane, is useful for evaluating heart failure and pulmonary hypertension. Therefore, many studies focused on end‐systolic pressure‐volume relationship (ESPVR) and end‐diastolic pressure‐volume relationship (EDPVR) derived from the PV loop as indices that can evaluate ventricular intrinsic contractility/dilatability independent of preload.

In order to apply the PV loop in clinical practice, it is desirable to estimate multiple PV loops using the conductance catheter in the ventricle and to change the preload by occlusion of the inferior vena cava or other means such as the multiple‐beat method (Grossman et al., 1977). However, the PV loop by the multiple‐beat method in practical use is limited because changing the preload of heart failure patients is difficult due to the risk of destabilizing the patient condition. Alternatively, the single‐beat method was proposed, which does not require preload manipulation to draw a PV loop (Sunagawa et al., 1980; Takeuchi et al., 1991). There were some studies on ESPVR, which demonstrated the PV loop and calculated parameters based on RHC results without using a conductance catheter (Sanz et al., 2012; Trip et al., 2013). However, there had been little established method for estimating the PV loop from RHC results. Meanwhile, a method using diastolic pressure values obtained from RHC and RV volume measured by CMR was proposed in 2011, which has been widely used in pulmonary hypertension patients (Rain et al., 2013). Conversely, there have been few studies on RV diastolic parameters such as diastolic stiffness coefficient (β) and end‐diastolic elastance (Eed) related to heart failure, possibly because several patients with heart failure have implanted devices, which are challenging to undergo CMR. On the other hand, there had been some reports about approximating ventricle properties using only pressure data of RHC. When we make a model of a theoretical nonejecting heartbeat, the maximum pressure can be calculated on a fitting of the early and late isovolumic contraction and relaxation ranges of the pressure waveform using sine function (Bellofiore et al., 2018; Colunga et al., 2023).

Therefore, we think a simple hemodynamic evaluation using one evaluation modality such as RHC might enhance the clinical usefulness and the information. In this study, we estimated the ESPVR and EDPVR using the RHC data alone through our algorithm based on the single‐beat method in heart failure patients. Moreover, we verified the correlation of the value of RV diastolic function such as β and Eed calculated by our method with the data calculated from the result of CMR. Using this method, we evaluated the diastolic function of right ventricle in patients with restrictive cardiomyopathy (RCM) and amyloidosis, which are representative of diastolic dysfunction.

## 2 METHODS

### 2.1 Patient selection

Heart failure patients admitted to our institution from January 2019 to May 2022 who underwent RHC within 10 days of CMR scans were retrospectively included. Patients with moderate or severe tricuspid, or pulmonary valve regurgitation, or congenital heart disease were excluded because such conditions render the premise of the isovolumic range of the single‐beat method invalid. The University of Tokyo Institutional Review Board reviewed and approved this study protocol conformed to the tenets of the Declaration of Helsinki (approval number: 2650). Informed consent was obtained in the form of an opt‐out selection on our web‐site.

### 2.2 Cardiac catheterization and other evaluations

After local anesthesia, a 7‐Fr ballooned Swan–Ganz catheter (Edwards Lifesciences) was inserted through the internal jugular vein and right atrial pressure, right ventricular pressure (RVP), pulmonary artery wedge pressure, and pulmonary artery pressure (PAP) were measured. RVP waveform was recorded with breath‐holding during the expiratory phase. Moreover, cardiac output (CO) and cardiac index (CI) were measured in all cases using thermodilution and the indirect Fick method. The stroke volume (SV) was obtained by dividing CO by heart rate.

Blood tests were performed at the time closest to the date of RHC. Echocardiography was also performed at the time closest to the date of RHC and normal echocardiographic parameters, including left ventricular ejection fraction (LVEF), E/A ratio (the ratio of peak velocity blood flow from left ventricular relaxation in early diastole [the E wave] to peak velocity flow in late diastole caused by atrial contraction [the A wave]), E/e’ ratio (the ratio of the E wave to early diastolic velocity of mitral annulus [e’]), right ventricular fraction area change (RVFAC), and tricuspid annular plane systolic excursion (TAPSE) were evaluated.

### 2.3 CMR imaging scan

All CMR images were obtained using 3 Tesla MRI system (IngeniaCX; Philips Healthcare). Cine sequences were obtained using ECG‐triggered, balanced turbo field echo (bTFE) sequence in left ventricular long axis images and short axis images from base to apex (echo time (TE) / repetition time (TR): 1.41 ms (four‐chamber view), 1.29 ms (two‐chamber view), 1.37 ms (short axis)/2.8 ms; flip angle (FA), 50°; slice thickness, 10 mm; FOV, 380 × 380 mm^2^; matrix, 128 × 128; reconstruction matrix, 256 × 256). RV volume was calculated using AZE virtual place system (AZE). To obtain the RV volume, endocardial borders of the RV were manually traced to calculate end‐systolic volume (ESV) and end‐diastolic volume (EDV).

### 2.4 Data analysis

#### 2.4.1 Overview of ESPVR and EDPVR

Based on single‐beating method of Sunagawa et al, ESPVR is defined by a straight line passing through the two points (ESV, end‐systolic pressure [ESP]) and (EDV, P_max_) (Sunagawa et al., 1980; Takeuchi et al., 1991; Trip et al., 2013). P_max_, ESV and EDV were described in later parts. End‐systolic elastance (Ees) is the slope of the ESPVR line, and arterial elastance (Ea) is defined by ESP/(EDV‐ESV).

EDPVR was defined as an exponential curve obtained using the following formula through the three points (0, 0), (ESV, begin‐diastolic pressure [BDP]), and (EDV, end‐diastolic pressure [EDP]) (Rain et al., 2013).

P = α (e^Vβ^ − 1) (P is pressure, α is a curve‐fitting constant, V is volume, and β is diastolic stiffness coefficient).

The β derived from this equation was defined as the diastolic stiffness.

The end‐diastolic elastance (Eed) was calculated using the following equation (Trip et al., 2015).

$\mathrm{Eed}=\alpha \cdot \beta \cdot e^{\left(\beta \cdot \mathrm{EDV}\right)}.$

#### 2.4.2 Calculation of P_max_

**FIGURE 1:**
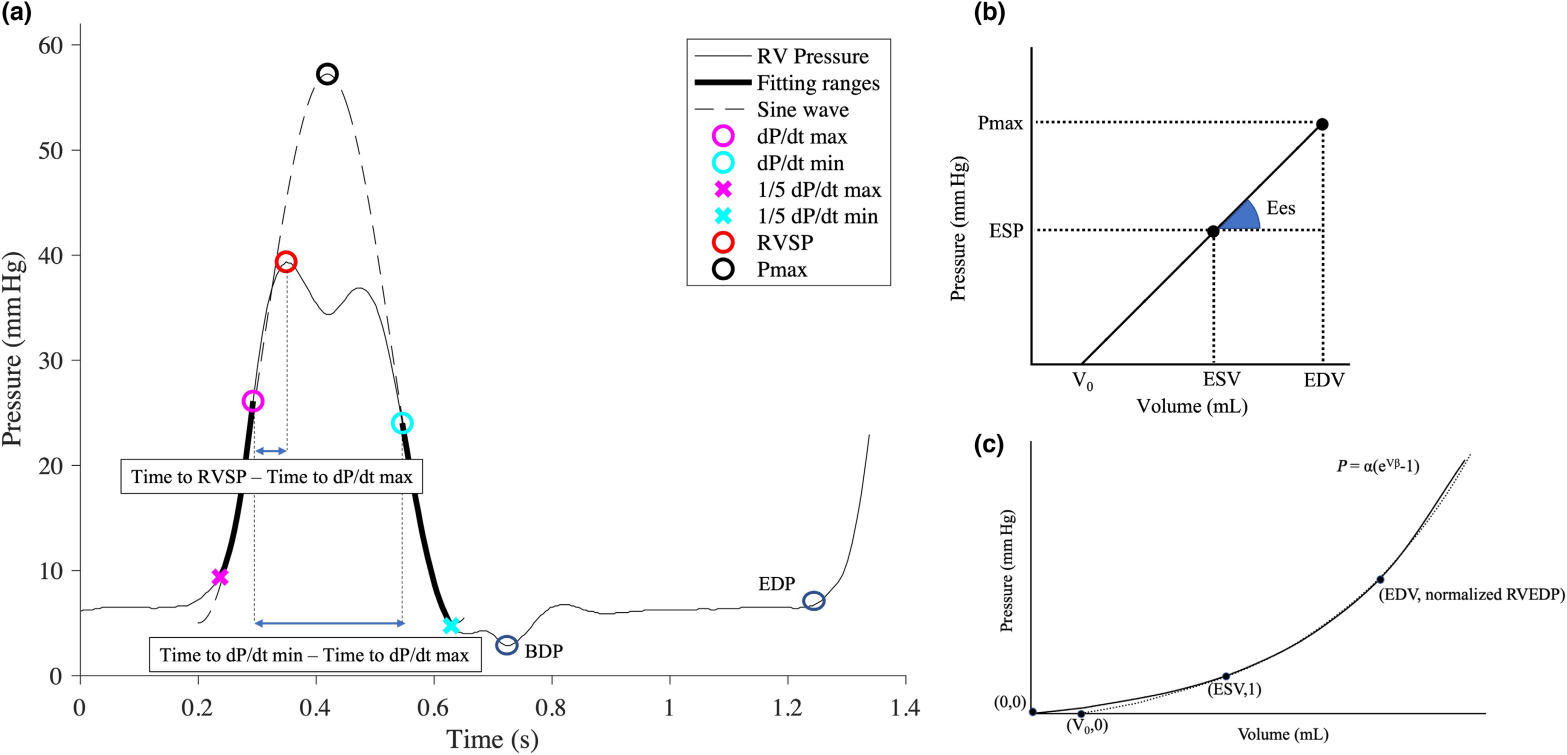
(a) illustrates the actual right ventricular pressure waveform, the points of each pressure measurement, and how the sine‐curve fitting is approximated. Sine‐curve fitting was performed during the isovolumic contraction (IC) and isovolumic relaxation (IR) periods. The IC period was defined between 1/5 dP/dt max (start of range; marked with a magenta cross) and dP/dt max point. IR period was defined between dP/dt min point and 1/5 dP/dt min (end of the range; marked with a cyan cross). The maximum value of the approximate sine curve was defined as P_max_. The two parameters (Time to RVSP—Time to dP/dt max, Time to dP/dt min—Time to dP/dt max) necessary to obtain ESP_modified_ are shown in (a). (b) illustrates the end‐systolic pressure‐volume relationship (ESPVR). The ESPVR is expressed as a linear line, where Ees is the slope of the line. We have assumed V_0_ to be zero and obtained ESV, EDV, and Ees from three parameters, ESP, P_max_, and SV. SV is the difference between EDV and ESV. (c) shows how the end‐diastolic pressure‐volume relationship (EDPVR) is approximated and β is calculated. The normalized RVEDP was defined as (EDP − BDP + 1). EDPVR was defined as an exponential function obtained using the following approximate formula through the three points (0, 0), (ESV, 1), (EDV, Normalized RVEDP) (Solid line). P = α(e^Vβ^−1) (P is pressure, V is volume). The value of β derived from this equation was defined as the diastolic stiffness coefficient. The dashed line is the approximated EDPVR line using the three points (V_0_, 0), (ESV, 1), (EDV, normalized RVEDP). BDP, begin‐diastolic pressure; EDP, end‐diastolic pressure; EDV, end‐diastolic volume; Ees, end‐systolic elastance; ESV, end‐systolic volume; RVEDP, right ventricular end‐diastolic pressure; RVSP, right ventricular systolic pressure; SV, stroke volume.

Clinical measurements of pressure waveforms were recorded digitally in a polygraph (Cath Lab RMC5000; Nihon Kohden) and comma‐separated values of the consecutive 10–15 RVP waveforms at a sampling rate of 1000 Hz were extracted. We analyzed 3 waveforms to be measured stably of them, using MATLAB software (R2021a, MathWorks, Natick, MA, USA Inc.). The range of IC and IR in the RVP waveform during one cycle was designated, and a sine‐curve fitting using the Levenberg–Marquardt least square algorithm was performed with the maximum value of the sine curve as P_max_. The range specifications for IC and IR were 1/5 dP/dt max to dP/dt max, and dP/dt min to 1/5 dP/dt min, respectively (Bellofiore et al., 2018). The P_max_ was calculated as the mean values of the 3 heartbeats (Figure 1a).

#### 2.4.3 Estimation of RV volume

Assuming that the ESPVR passes through (0, 0), the ESV and EDV were calculated from the three values of P_max_, ESP, and SV.

For ESP, we used ESP_mPAP_ using mean pulmonary artery pressure (mPAP) and ESP_modified_ derived from the following equation (Wright et al., 2020).

ESP_modified_ = mPAP + [(systolic pulmonary artery pressure (sPAP) − mPAP) × (time to RVSP − time to dP/dt max)/(time to dP/dt min − time to dP/dt max)] (Figure 1a).

Furthermore, two SV values were utilized: SV_Thermo_ using the thermodilution method and SV_Fick_ using the indirect Fick method (Figure 1b). To calculate β and Eed, we investigated about the correlation between the ESV and EDV obtained by the methods mentioned above and those from the CMR evaluation (Figure 1c), and determined the best ESP and SV parameters for estimating RV volume.

#### 2.4.4 Estimation of EDPVR, β, and Eed

EDPVR was defined as an exponential curve obtained using the following formula through the three points (0, 0), (ESV, 1), and (EDV, normalized RVEDP) (Rain et al., 2013). We used 1 instead of BDP and normalized RVEDP instead of EDP to minimize measurement errors caused by the positioning of the catheter. Normalized RVEDP is defined as (right ventricular end‐diastolic pressure [RVEDP] − right ventricular begin‐diastolic pressure [RVBDP] + 1). Using the normalized RVEDP and the ESV and EDV calculated in 2.4.3, estimated β and Eed were obtained from the approximate formula for EDPVR. We also calculated β (CMR) and Eed (CMR) by using ESV and EDV derived from CMR instead of those calculated in 2.4.3. We investigated about the correlation between the ESV and EDV from the CMR evaluation with those obtained by the method mentioned above (Figure 1c).

### 2.5 Statistical analysis

Data are expressed as means with standard deviations or medians with interquartile ranges. We used the Shapiro–Wilk test to assess the normality of data distribution. We also used the Student's t‐test or Mann–Whitney *U* test to compare the continuous variables, whereas Fisher's exact test was used to compare the categorical variables. The confidence interval was 95%, and *p* ≤ 0.05 was considered statistically significant. Baseline data were analyzed using paired t‐test or Wilcoxon signed‐rank test. Pearson's test was used for the linear correlation analysis. All statistical analyses were performed using JMP software (version 14.2; SAS Institute).

## 3 RESULTS

### 3.1 Baseline characteristics of the participants

A total of 53 patients were enrolled in this study from January 2019 to May 2022. Seven patients were excluded because of the unclear pressure waveforms, inability to perform a stable pressure assessment due to large respiratory variability, and difficulty in CMR volume assessment due to arrhythmia. The remaining 46 patients with heart failure comprised 27 with dilated cardiomyopathy (DCM), 6 with amyloidosis including amyloid light chain and transthyretin amyloidosis, 5 with hypertrophic cardiomyopathy, 2 with drug‐induced cardiomyopathy, 2 with RCM, 1 with ischemic cardiomyopathy, 1 with cardiac sarcoidosis, 1 with left ventricular aneurysm of unknown cause and 1 with left ventricular noncompaction. The clinical characteristics of patients are presented in Table 1.

**TABLE 1:** Patient characteristics.

Characteristics	*n* = 46
Age, years	53.9 ± 19.8
Male, *n* (%)	30, 65.2%
BMI (kg/m^2^)	22.9 [19.9–26.1]
Hb (g/dL)	14.0 ± 2.0
Cre (mg/dL)	0.83 [0.74–0.96]
BNP (pg/mL)	170.0 [76.2–404.0]
LVEF (%)	33.5 [22.0–50.3]
mRAP (mm Hg)	5.0 [2.0–6.0]
RVSP (mm Hg)	27.5 [21.8–33.0]
RVEDP (mm Hg)	5.0 [3.0–7.3]
Normalized RVEDP (mm Hg)	5.0 [4.0–7.0]
mPAP (mm Hg)	16.0 [11.8–22.3]
mPAWP (mm Hg)	10.0 [6.8–13.0]
HR (/min)	68.1 ± 11.8
CI, Thermo (liter/min/m^2^)	2.36 [2.03–2.73]
CI, Fick (liter/min/m^2^)	1.86 [1.70–2.14]
RVEDV (mL)	141.9 [104.0–163.0]
RVESV (mL)	75.3 [53.9–103.5]

*Note*: Data are expressed as means ± standard deviations or medians (interquartile ranges).

Abbreviations: BMI, body mass index; BNP, B‐type natriuretic peptide; CI, cardiac index; Cre, Creatinine; Fick, estimated oxygen uptake Fick method; Hb, hemoglobin; HR, heart rate; LVEF, left ventricular ejection fraction; mPAP, mean pulmonary artery pressure; mPAWP, mean pulmonary artery wedge pressure; mRAP, mean right atrium pressure; RVEDP, right ventricular end‐diastolic pressure; RVEDV, right ventricular end‐diastolic volume; RVESV, right ventricular end‐systolic volume; RVSP, right ventricular systolic pressure; Thermo, Thermodilution method.

### 3.2 Correlation between RV parameters exemplified by RVP waveforms and CMR

First, we calculated the estimated RV volume using two methods for estimating stroke volumes (thermodilution and indirect Fick method [SV_Thermo_, SV_Fick_]) and two methods for estimating ESPs (ESP_mPAP_ and ESP_modified_). We investigated the correlation between the estimated RV volume and that determined from CMR imaging.

**FIGURE 2:**
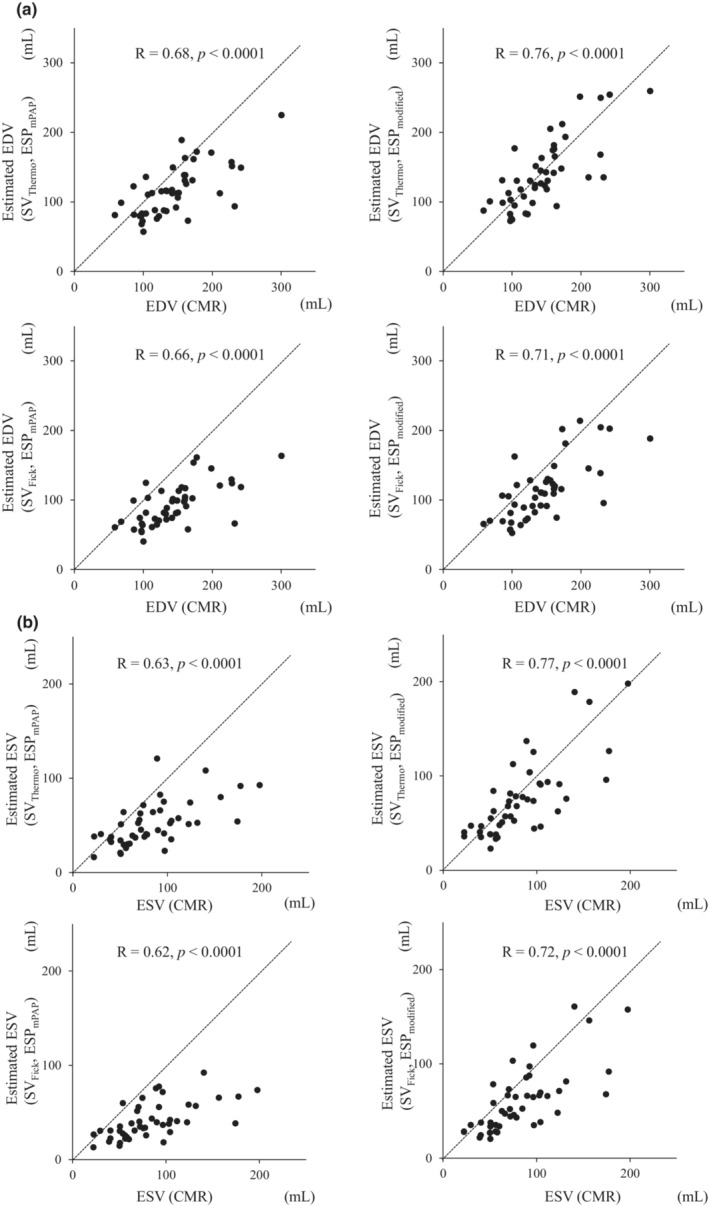
(a) Scatter plot showing the relationship of RVEDV measured by CMR (x‐axis) and that calculated by our method (y‐axis). (b) Scatter plot of the RVESV measured by CMR (x‐axis) and that calculated by our method (y‐axis). The dotted lines represent straight lines with y = x. In our method, we calculated the estimated right ventricular volume using two methods for estimating stroke volumes (thermodilution and indirect Fick's method [SV_Thermo_, SV_Fick_]) and two methods for estimating end‐systolic pressures (ESP_mPAP_ and ESP_modified_). CMR, cardiac magnetic resonance imaging; RVEDV, right ventricular end‐diastolic volume; RVESV, right ventricular end‐systolic volume.

As shown in Figure 2, all four estimated EDVs and ESVs were correlated with those derived from CMR, with the methods using SV_Thermo_ and ESP_modified_ showing the best correlation and consistency (EDV: R = 0.76, *p* < 0.0001, ESV: R = 0.77, *p* < 0.0001). Therefore, we adopted this method to estimate EDVs and ESVs using the SV_Thermo_ and ESP_modified_ for further evaluation.

**FIGURE 3:**
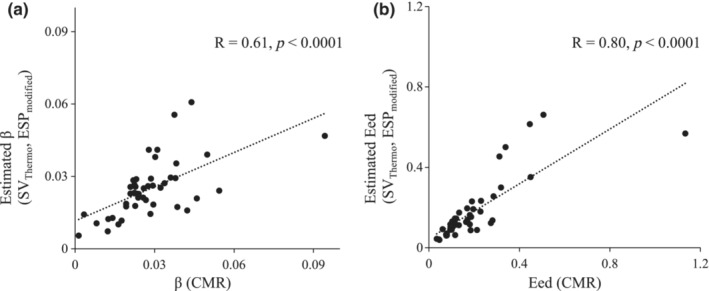
(a) Scatter plot showing the relationship of β calculated by EDV and ESV derived from CMR (x‐axis) and that calculated by our method (y‐axis). (b) Scatter plot of Eed calculated by EDV and ESV derived from CMR (x‐axis), Eed calculated by our method (y‐axis). In our method, we calculated each estimated right ventricular volume using stroke volumes derived from the thermodilution method (SV_Thermo_) and substituting ESP_modified_ for end‐systolic pressure. CMR, cardiac magnetic resonance imaging; EDV, end‐diastolic volume; Eed, end‐diastolic elastance; ESV, end‐systolic volume; β, diastolic stiffness coefficient;

The estimated β and Eed were calculated using EDVs and ESVs, which were determined by the above method. We compared β (CMR) and Eed (CMR) using ESV and EDV obtained from CMR examinations with the estimated β and Eed by our method (Figure 3). Significant correlations were noted between both parameters, and a strong correlation was observed for Eed (β: R = 0.61, *p* < 0.0001, Eed: R = 0.80, *p* < 0.0001).

### 3.3 Comparison of β and Eed in different disease groups

**FIGURE 4:**
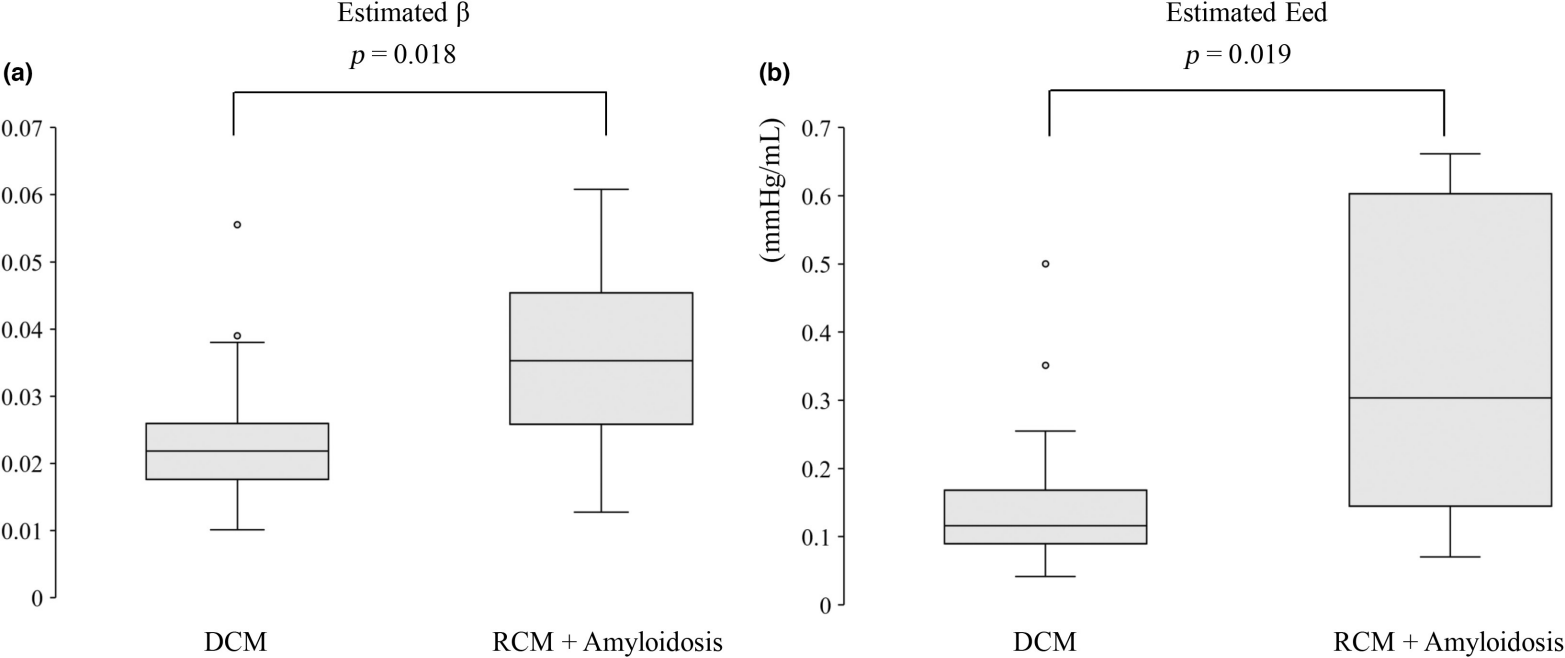
(a) shows a comparison of box plots of β calculated by our method between the DCM and RCM with amyloidosis groups. (b) shows a comparison of box plots of Eed calculated by our method between the DCM and RCM with amyloidosis groups. CMR, cardiac magnetic resonance imaging; DCM, dilated cardiomyopathy; Eed, end‐diastolic elastance; RCM, restrictive cardiomyopathy; β, diastolic stiffness coefficient.

**FIGURE 5:**
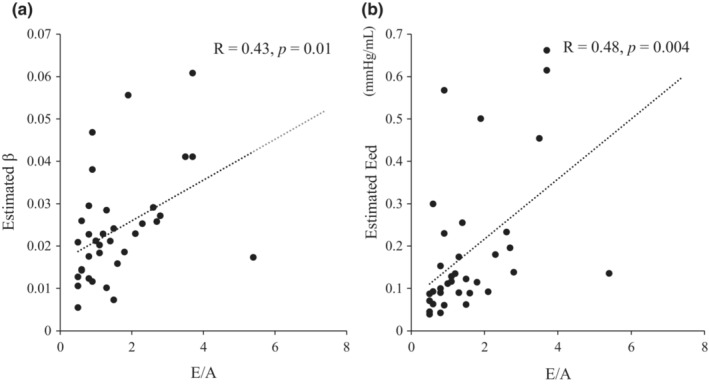
The relationship between E/A and estimated β or estimated Eed. E/A is one of the parameters of left ventricular diastolic function measured by echocardiography. There are significant correlations between E/A and both estimated β and Eed. E/A, the ratio of peak velocity blood flow from left ventricular relaxation in early diastole (the E wave) to peak velocity flow in late diastole caused by atrial contraction (the A wave).

A comparison between the DCM group (*n* = 27) and the RCM with the amyloidosis group, which was characterized by marked diastolic dysfunction (*n* = 8), was performed (Table 2). A significant difference was noted in Ees and RVEF measured by CMR between the two groups in systolic function. However, no significant difference was noted between the two groups for TAPSE and RVFAC, which are commonly used parameters of systolic function on echocardiography. Regarding diastolic function, no difference was observed in E/A and septal E/e', which is most frequently assessed by echocardiography. However, the β and Eed calculated by our method were significantly different between the two groups. Figure 4 shows a comparison of the box plots of the β and Eed calculated by our method, indicating a more significant sensitivity of our method than the existing diastolic parameters. In addition, the β and Eed calculated by our method were finely correlated with E/A on echocardiography (β: R = 0.43, *p* = 0.010, Eed: R = 0.48, *p* = 0.0035) (Figure 5).

**TABLE 2:** Comparison of parameters between DCM and RCM + amyloidosis group.

	DCM (*n* = 27)	RCM + amyloidosis (*n* = 8)	*p*‐value
LVEF (%)	25.0 [20.0–35.0]	53.5 [41.5–56.0]	<0.001*
E/A	1.30 [0.85–2.00] (*n* = 21)	2.20 [0.73–3.70] (*n* = 6)	0.579
E/e’	12.90 [10.40–19.40]	15.25 [9.15–21.05]	0.844
TAPSE (mm)	15.5 [14.0–18.8] (*n* = 20)	16.0 [13.0–19.0] (*n* = 7)	0.956
RVFAC (%)	34.0 [30.0–40.0] (*n* = 23)	33.0 [27.0–43.0] (*n* = 7)	1.000
RVEF (CMR, %)	40.7 [29.3–44.4]	51.7 [41.6–57.7]	0.013*
RVEDP (mm Hg)	6.0 [4.0–7.0]	5.5 [3.5–16.0]	0.579
Normalized RVEDP (mm Hg)	5.0 [4.0–6.0]	7.5 [5.0–11.8]	0.049*
PVR (Wood)	1.42 [1.05–2.62]	2.43 [1.12–3.10]	0.263
PAPi	3.40 [2.67–5.00]	4.10 [2.49–7.25]	0.623
RVSWI (gm/beat/m^2^)	6.19 [4.81–8.09]	7.35 [4.80–10.55]	0.377
Ees (mm Hg/mL)	0.24 [0.17–0.33]	0.49 [0.32–0.62]	0.002*
Ea (mm Hg/mL)	0.29 [0.22–0.50]	0.44 [0.34–0.61]	0.141
Ees/Ea	0.79 [0.57–1.01]	1.10 [0.84–1.22]	0.024*
Estimated EDV (mL)	147.8 [126.5–193.2]	105.6 [85.4–127.6]	0.010*
Estimated ESV (mL)	90.7 [67.7–112.4]	51.3 [39.5–70.3]	0.006*
Estimated β	0.023 [0.018–0.028]	0.035 [0.026–0.045]	0.018*
Estimated Eed (mm Hg/mL)	0.116 [0.090–0.174]	0.303 [0.145–0.603]	0.019*
β (CMR)	0.026 [0.022–0.032]	0.029 [0.021–0.042]	0.623
Eed (CMR) (mm Hg/mL)	0.133 [0.100–0.212]	0.249 [0.116–0.491]	0.103

*Note*: PAPi = (systolic pulmonary artery pressure − diastolic pulmonary artery pressure)/mean right atrial pressure. RVSWI = (stroke volume/body surface area) × (mean pulmonary capillary wedge pressure − mean right atrial pressure).

Abbreviations: DCM, dilated cardiomyopathy; RCM, restrictive cardiomyopathy; LVEF, left ventricular ejection fraction; E, early mitral flow velocity; A, peak velocity flow in late diastole caused by atrial contraction; e, early diastolic velocity of the septal mitral annulus; TAPSE, tricuspid annular plane systolic excursion; RVFAC, right ventricular fractional area change; RVEF, right ventricular ejection fraction; PVR, pulmonary vascular resistance; PAPi, pulmonary artery pulsatility index; RVSWI, right ventricular stroke work index; Ees, end‐systolic elastance; Ea, arterial elastance.

* *p* < 0.05.

## 4 DISCUSSION

In this study, we demonstrated a method for estimating RV diastolic function, β and Eed, solely based on the RHC results, including pressure waveforms. Physiologically, the PV loop has been studied to evaluate ventricular contractility and diastolic function independent of preload. The method in this study was derived from a deeper exploration and application of the single‐beat method using RHC data only.

**FIGURE 6:**
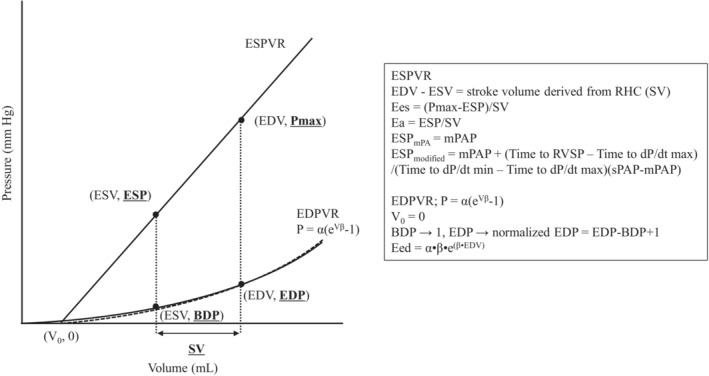
The overview of estimating PV loop from RHC data. In our evaluation method, each important parameter, which is required to estimate PV loop is presented in the square. BDP, begin‐diastolic pressure; Ea, arterial elastance; EDP, end‐diastolic pressure; EDPVR, end‐diastolic pressure‐volume relationship; EDV, end‐diastolic volume; Eed, end‐diastolic elastance; Ees, end‐systolic elastance; ESP, end‐systolic pressure; ESPVR, end‐systolic pressure‐volume relationship; ESV, end‐systolic volume; mPAP, mean pulmonary artery pressure; PV, pressure‐volume; RHC, right heart catheterization; RVSP, right ventricular systolic pressure; sPAP, systolic pulmonary artery pressure; SV, stroke volume; β, diastolic stiffness coefficient.

In this study, we elaborated the simple way to evaluate diastolic RV function using only RHC data (Figure 6). Indeed, if it is an evaluation that actually combines a plurality of examinations, validity will be greatly reduced due to differences in the dates of examinations, appropriateness of subjects in each examination. In addition, echocardiography has limitations in the evaluation of right heart function, such as difficulty in visualization. It is comparatively difficult for computed tomography or CMR to estimate intracardiac pressure. In order to derive clinical usefulness, it seems necessary to pursue the simplicity and convenience of the evaluation method and we focused the utility of RHC.

The single‐beat method is based on the sine‐curve fitting of ventricular pressure waveforms in the IC and IR ranges and the estimation of P_max_ derived from sine‐curve fitting (Sunagawa et al., 1980; Takeuchi et al., 1991). This method can predict ESPVR from a single PV loop without changing the preload and has been studied in the left ventricle. Some have argued that the single‐beat method cannot be applied to the RV owing to its crescent shape and asymmetric contraction and dilation patterns (Maughan et al., 1979). However, in 2003, Brimioulle et al. reported that the single‐beat method could be applied to the RV appropriately (Brimioulle et al., 2003), and PV loop estimation in the RV has been used in clinical research since then.

In terms of systolic function derived from the PV loop, the usefulness for methods to estimate Ees and Ea from RHC alone had been reported in patients with pulmonary hypertension and heart failure (Richter et al., 2020; Schmeißer et al., 2021; Tello et al., 2019).

Like the systolic function parameter derived from the PV loop, the parameter of the diastolic function derived from the PV loop was also reported to have clinical application. A report published in 2011 on the evaluation of EDPVR without conductance catheters became the standard method (Rain et al., 2013). This method requires volumetric evaluation by CMR, and several reports about pulmonary hypertension were documented. The value of β correlates with transplant‐free survival in patients with pulmonary hypertension (Vanderpool et al., 2015), and an Eed of >0.53 is associated with poor prognosis (Trip et al., 2015). Moreover, an elevated Eed correlates with myocardial T1 mapping parameters on CMR (Tello et al., 2019). However, few studies have evaluated EDPVR in heart failure patients.

In this study, the methods are based on approximations of various parameters. The first is estimating the range of IC and IR when performing sine‐curve fitting. The points dP/dt max and dP/dt min are commonly used for the end of the IC and the beginning of the IR, respectively. On the other hand, the beginning of IC is sometimes defined as “the start of contraction” and the end of IR as “the start of dilation,” which often makes the definitions ambiguous, resulting in an interobserver difference. A recent report on sine‐curve fitting claims that the interobserver difference in P_max_ can be minimized by setting the start of IC and the end of IR as 1/5 dP/dt max and 1/5 dP/dt min, respectively (Bellofiore et al., 2018). Therefore, this approach was adopted in this study.

The second point is the ESP estimation. In previous years, mPAP has been commonly used for ESP (Chemla et al., 1996); however, the discrepancy between ESP and mPAP was recently reported to become greater as the mPAP increases (Philip & Chesler, 2018; Tello et al., 2018). A new method to estimate ESP was proposed for the problem, as described in the Methods section (Wright et al., 2020). This method adds a fixed percentage of the difference between mPAP and sPAP to mPAP, according to the arrival time of the peak RV pressure waveform. The formula is based on the phenomenon that the peak point of RV pressure waveform is prone to be later, and the discrepancy between ESP and mPAP increases as pulmonary hypertension progresses. We used two methods of ESP estimation: the conventional method and this new method.

The third point is the V_0_ estimation. V_0_ is the X‐intercept value when P = 0 in the straight line of ESPVR and is defined as the volume left in the ventricle after contraction against zero loads, often called dead volume (Trip et al., 2013). In this study, the value of V_0_ is required to calculate ESV, EDV, and diastolic parameters. Although there are some difficulties in calculating V_0_, a simple way of setting V_0_ as 0 has been verified in the calculation of RVEF and diastolic function parameters, such as β and Eed, in the cases of pulmonary hypertension (Heerdt et al., 2020; Vanderpool et al., 2019). We proceeded with our analysis based on these backgrounds by setting the V_0_ to 0. When we calculated the value of both β and Eed using V_0_, which was estimated using CMR, there was a strong correlation in the value of both β and Eed (β: R = 0.99, *p* < 0.0001, Eed: R = 1.00, *p* < 0.0001) between the current method and the method using V_0_ derived from CMR data (Supplemental Method and Figure S1—Data S1), which demonstrated the validity of V_0_ as 0.

Under these assumptions, a significant correlation was observed between the RV volume calculated by CMR and the RV volume calculated by our method, regardless of the method used to estimate ESP (ESP_mPAP_, ESP_modified_) or calculate SV (SV_Thermo_, SV_Fick_). Furthermore, the use of ESP_modified_ and SV_Thermo_ was correlated more closely with the data exemplified by CMR. The selection of the thermodilution method in SV calculation was consistent with the previous study, which was more reflective of all‐cause mortality than the Fick method (Opotowsky et al., 2017).

To assess the clinical utility of various parameters obtained by our method, we compared DCM patients with combined RCM and amyloidosis patients. The low RVEF value in the DCM group corresponded well, with a significant difference in Ees. However, none of the parameters of RV contractility on echocardiography showed significant differences. By contrast, β and Eed tended to be higher in the RCM and amyloidosis groups, which suggests that our method can assess diastolic function more sensitively. We also found the correlation between β or Eed and E/A on echocardiography. Indeed, the results may differ from the differences in the evaluation modalities that should be explored in the future. In addition, the result demonstrated RV diastolic function was also severely impaired in patients with RCM and amyloidosis, similar with diastolic dysfunction in left ventricle. We think the difference in diastolic property of RV seem to be due to myocyte intrinsic difference (Jung et al., 2022). This should be investigated further in the future.

Using our method, β and Eed can be obtained in all patients who undergo RHC, which may increase the number of cases. This method can also be used for heart failure patients with difficulty undergoing CMR, such as patients with hemodynamic instability or left ventricular assist device (LVAD) implantation. It has the potential to provide a new risk stratification method for predicting right heart failure, especially for patients with LVAD implantation, by evaluating the right heart function using our method.

This study had some limitations. First, the RV volume manually measured by CMR may have some variation. However, CMR is regarded as one of the most reliable methods for evaluating RV volume (Freling et al., 2013; Sardanelli et al., 2008). Moreover, we evaluated RV volume along the short axis of the CMR. It has been reported that volume assessment in the short axis is as accurate and reliable as in a four‐chamber view, with less interobserver difference (Couto et al., 2020). Furthermore, good correlation between CMR‐derived SV and thermodilution‐derived SV (R = 0.68, *p* < 0.0001), supporting the validity of CMR volumetric evaluation.

Second, we recruited the cases that underwent RHC within 10 days of CMR, and the actual volume may have changed due to heart failure treatment.

The third limitation was the participant selection criteria in this study. We excluded the severe heart failure cases because CMR cannot be performed until the heart failure status is stabilized to a certain extent. Therefore, whether this technique can be applied to severe heart failure patients is uncertain. Moreover, it is expected that as the severity of pulmonary hypertension increases, RV volume and V_0_ also increase (Trip et al., 2013). Therefore, the question remains whether this method can be used to evaluate diastolic function in severe heart failure patients. However, the larger the RV volume is, the greater the probability of having moderate or severe tricuspid regurgitation, which cannot apply the single‐beat method. Therefore, many cases with large RV volumes with significant V_0_ are likely to meet the exclusion criteria and are not eligible for our method. We should be careful about the appropriate indication for our evaluation in terms of the estimation of V_0_ = 0. In addition, the selection of method of thermodilution or ESP modified was not verified in robust way; therefore, the appropriateness of these selections should be also verified using other patients populations. The method of sine/cos wave fitting was highly empirical, however, close correlation between CMR‐derived RV volume and RV volume calculated by our method finely supported the validity of the empirical calculation.

In order to confirm the usefulness of this our method, we confirmed that the RV diastolic function of the RCM and amyloid group was actually significantly impaired. However, since there is no easily performed available evaluation method of RV diastolic function at this time, it is difficult to conduct a test for verification of our evaluation method in a strict way. In the future, it is thought that the most important method for confirming the usefulness of our evaluation of diastolic function will be to show the relationship of the value with the heart failure status in various clinical situations. In addition, there might be some possibilities that volume status could make some impacts on the value of diastolic properties because the volume status in heart failure was thought to vary.

## 5 CONCLUSIONS

We studied the estimation of RV volume and diastolic function parameters from RHC alone based on the single‐beat method and it correlated well with those calculated by CMR. Our findings suggested that the diastolic function parameters derived from our method may reflect actual RV diastolic function. By the method, RV dysfunction was finely identified in patients with RCM and amyloidosis.

## AUTHOR CONTRIBUTIONS

Yoshitaka Isotani involved in conceptualization, methodology, data curation, validation, writing original draft, and review and editing. Eisuke Amiya involved in conceptualization, methodology, validation, data curation, and review and editing. Tatsuya Suzuki involved in methodology and validation. Masaru Hatano and Issei Komuro involved in supervision. Hiroyuki Kiriyama, Masae Uehara, Junichi Ishida, Shun Minatsuki, Hiroki Yagi, Akihito Saito, Masaki Tsuji, Chie Bujo, Koichi Narita, Satoshi Ishii, Nobutaka Kakuda, Genri Numata, Takanobu Yamada, and Takahiro Kurihara involved in data curation. All authors contributed to the article and approved the submitted version.

## FUNDING INFORMATION

This work was supported by the Ministry of Education, Culture, Sports, Science and Technology of Japan through Grant‐in‐Aid 21K08047 (to EA).

## CONFLICT OF INTEREST STATEMENT

EA belongs to the Department, endowed by NIPRO‐Corp, Terumo‐Corp., Senko Medical‐Instrument‐Mfg., Century‐Medical, Inc., ONO‐pharmaceutical‐Co., Ltd. Medtronic‐JAPAN Co., Ltd, Nippon‐Shinyaku Co., Ltd, Mochida Pharmaceutical Co., Boehringer Ingelheim Pharmaceuticals Inc., Abiomed‐Inc, AQuA‐Inc, Fukuda‐Denshi Co., Ltd, and Sun‐Medical‐Technology‐Research Corp. EA received research fund from Bristol‐Myers Squibb Co.

## Supporting information


Data S1.
