# Supplementary material for: A new assessment method for right ventricular diastolic function using right heart catheterization by pressure‐volume loop
Source: Physiol Rep. 2023 Jul 2;11(13):e15751. doi: 10.14814/phy2.15751 (PMC10315326; doi:10.14814/phy2.15751)
Supplement: Supplementary file 1 — Data S1. [file PHY2-11-e15751-s001.pdf]

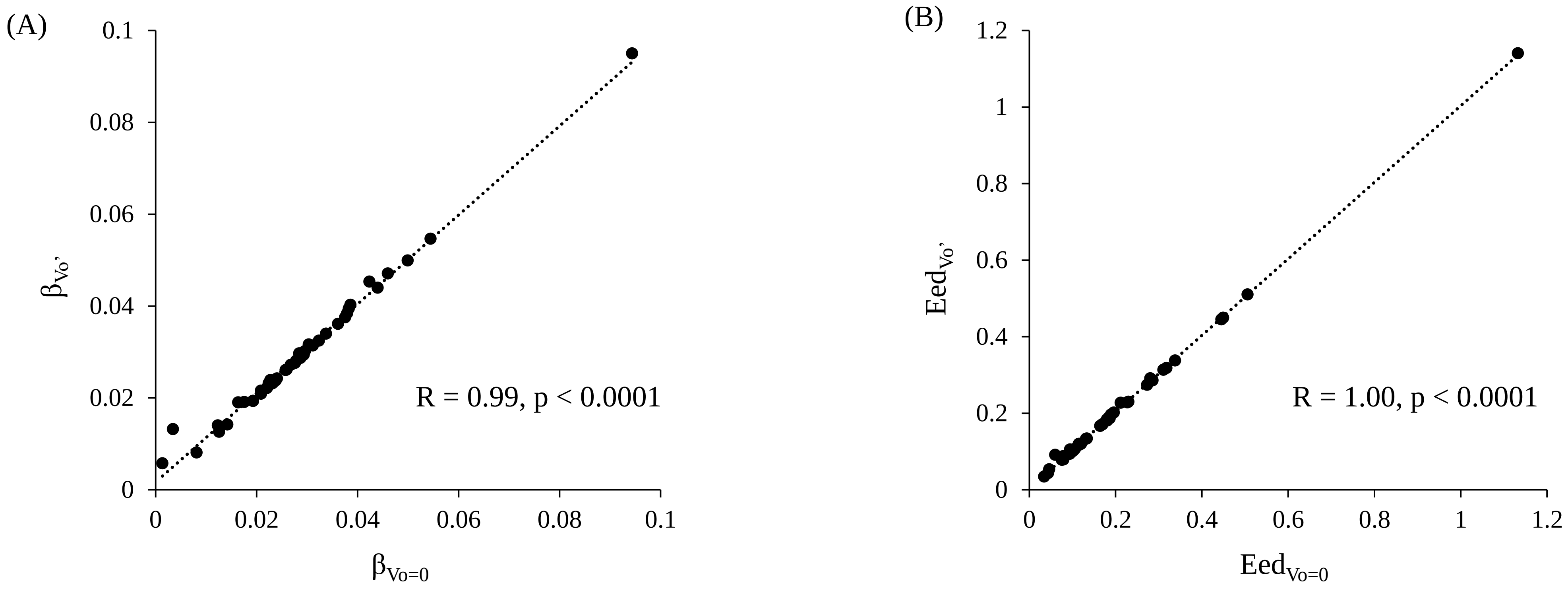

Supplemental Method

We examined the effect of different V_0 in determining β, Eed as described previously [25]. We derived V_0' by calculating the X-intercept of the ESPVR line passing through (ESV obtained from CMR, $ESP_{modified}$), (EDV obtained from CMR, Pmax) (Figure 1B). The average V_0' was 12.8 mL (IQR: − 5.8–31.4 mL). The β and Eed were calculated under the assumption that $V_0 = 0$ ($\beta_{Vo=0}$ and $Eed_{Vo=0}$) , which were derived from estimated EDPVR using [(0,0), (ESV obtained from CMR, 1), (EDV obtained from CMR, normalized RVEDP)] described in the Methods section. We also calculated $\beta_{Vo'}$ and $Eed_{Vo'}$ by using (V_0', 0) instead of (0, 0) when approximating EDPVR (Figure 1C). We compared $\beta_{Vo=0}$ and $Eed_{Vo=0}$ with $\beta_{Vo'}$ and $Eed_{Vo'}$ to examine the impact of different V_0 on calculating β and Eed.

Supplemental Figure 1; (A) Scatter plot of $\beta_{Vo=0}$ and $\beta_{Vo'}$ (B) Scatter plot of $Eed_{Vo=0}$ and $Eed_{Vo'}$ Significant correlations were obtained for both β and Eed (β: R = 0.99, $p < 0.0001$, Eed: R = 1.00, $p < 0.0001$).

β, diastolic stiffness coefficient; Eed, end-diastolic elastance; EDPVR, end-diastolic pressure-volume relationship; ESV, end-systolic volume; CMR, cardiac magnetic resonance imaging; RVEDP, right ventricular end-diastolic pressure.
